# Morpholino-mediated SOD1 reduction ameliorates an amyotrophic lateral sclerosis disease phenotype

**DOI:** 10.1038/srep21301

**Published:** 2016-02-16

**Authors:** M. Nizzardo, C. Simone, F. Rizzo, G. Ulzi, A. Ramirez, M. Rizzuti, A. Bordoni, M. Bucchia, S. Gatti, N. Bresolin, G. P. Comi, S. Corti

**Affiliations:** 1 Dino Ferrari Centre, Neuroscience Section, Department of Pathophysiology and Transplantation (DEPT), University of Milan, Neurology Unit, IRCCS Foundation Ca’ Granda Ospedale Maggiore Policlinico, Milan, Italy; 2 Centro di Ricerche Chirurgiche Precliniche, IRCCS Foundation Ca’ Granda Ospedale Maggiore Policlinico; Department of Pathophysiology and Transplantation (DEPT), University of Milan, 20122 Milano, Italy

## Abstract

Neurotoxicity due to the accumulation of mutant proteins is thought to drive pathogenesis in neurodegenerative diseases. Mutations in superoxide dismutase 1 (SOD1) are linked to familial amyotrophic lateral sclerosis (fALS); these mutations result in progressive motor neuron death through one or more acquired toxicities. Interestingly, SOD1 is not only responsible for fALS but may also play a significant role in sporadic ALS; therefore, SOD1 represents a promising therapeutic target. Here, we report slowed disease progression, improved neuromuscular function, and increased survival in an *in vivo* ALS model following therapeutic delivery of morpholino oligonucleotides (MOs) designed to reduce the synthesis of human SOD1. Neuropathological analysis demonstrated increased motor neuron and axon numbers and a remarkable reduction in astrogliosis and microgliosis. To test this strategy in a human model, we treated human fALS induced pluripotent stem cell (iPSC)-derived motor neurons with MOs; these cells exhibited increased survival and reduced expression of apoptotic markers. Our data demonstrated the efficacy of MO-mediated therapy in mouse and human ALS models, setting the stage for human clinical trials.

Amyotrophic lateral sclerosis (ALS) is a fatal neurological disease characterized by the degeneration and loss of upper and lower motor neurons (MNs),which leads to paralysis and death within 3–5 years of diagnosis^1^. Currently, there is no effective treatment for this disease^2^. The majority of cases of ALS have no clear genetic linkage and are referred to as sporadic (sALS), while 10% of cases are familial (fALS)^3^. Disease-causing mutations in various genes have been identified^3^. Mutations in the gene encoding for Cu/Zn superoxide dismutase 1 (SOD1) are relatively frequent^4^, accounting for 15% of sALS. Mutations in the *SOD1* gene are linked to 20% of ALS cases. In these cases, the resulting progressive MN death is likely caused by one or more mutated SOD1-related toxicities, as revealed by studies of transgenic rodent models^5^,^6^,^7^,^8^.

Recent evidence supports SOD1 as a toxic factor not only in fALS but also in sALS^9^. Indeed, changes in oxidation, demetallation, and other types of post-translational modifications are able to induce aberrant conformations of wild-type (WT) SOD1, which eventually lead to its acquisition of toxic functions comparable to those of fALS-associated SOD1 variants^10^,^11^,^12^. Due to a series of conformation-specific antibodies, SOD1 has been detected in spinal cord samples from ALS patients and SOD1 rodent models in an altered/abnormal conformation, conventionally referred to as misfolded, which may account for its inherent toxic nature^10^,^13^,^14^. Misfolded “mutant-like” forms of WT SOD1 have also been found in human post-mortem tissue from patients affected by sALS, suggesting a concrete pathogenetic role for these SOD1 variants^10^,^13^. This finding, along with reports that a reduction of both WT and mutant SOD1 in astrocytes derived respectively from sALS and fALS patients decreased astrocyte-derived toxicity towards MNs^15^, provides strong evidence for a pathogenic role of WT SOD1 in sALS.

Mutant SOD1 protein induces a pathogenic phenotype when specifically expressed in MNs^16^; however, it also contributes to disease onset and early disease progression when it is expressed in microglia^16^, astrocytes^15^,^17^, and oligodendrocytes^18^. These findings indicated that ALS is also a non-cell autonomous disease.

Therefore, the ability to interfere with toxic SOD1 overexpression and its misfolded form may lead to strong advances in the treatment of both the familial and sporadic forms of ALS. Antisense oligonucleotides (ASOs) seem to be a promising tool for achieving this goal. ASOs are nucleic acid analogs designed to trap RNAs by binding in a particular place and interfering with a specific biological process, such as splicing or translation^19^. They represent a promising therapeutic strategy for the treatment of various human disorders and are currently being tested in clinical trials. In preclinical and clinical trials, two different chemical variants of ASOs have been studied: the 2′-O-methyl-modified phosphorothioate oligonucleotides or its more stable variant, 2′-O-(2-methoxyethyl)-modified phosphorothioate oligonucleotides; and the morpholino oligomers (MOs). MOs are analogs of natural nucleic acids in which the phosphorothioate-ribose backbone is replaced with a phosphorodiamidate-linked morpholine backbone that is refractory to metabolic degradation^20^. A Phase 1, randomized, first-in-human study with an ASO (2OMePS, ISIS 333611) against SOD1, which was delivered intrathecally to patients with SOD1 fALS, has been completed, demonstrating the safety and tolerability of this approach^21^.

Interestingly, impressive therapeutic rescue has been observed using MOs in another genetic motor neuron disease, spinal muscular atrophy (SMA), which is caused by mutations in the survival motor neuron 1 gene^22^,^23^,^24^. Recently, we confirmed this result by combining local and systemic administration of a 25-nt MO sequence (MO-10–34) in transgenic SMA mice. We effectively increased the expression of full-length SMN and observed robust neuromuscular improvement. Furthermore, in some cases, the survival rescue phenotype was essentially indistinguishable from that of healthy heterozygous mice^25^. While a direct comparison between methoxyethyl (MOEs) and MOs has not yet been performed for MNDs, it is likely that MOs will present greater advantages than MOEs. MOs exhibit low toxicity and high stability, a wide distribution in tissues, and in clinical trials, such as those for Duchenne Muscular Dystrophy, were associated with encouraging results that seem superior to those obtained using other ASOs^19^,^26^. While the safety and feasibility of the use of MOEs to reduce SOD1 have already been demonstrated in a phase 1 clinical trial^21^, the use of MOs has not yet been explored in the ALS field.

Here, we investigated MO sequences targeting SOD1 in rodent and human fALS models and demonstrated their efficacy in significantly reducing SOD1 levels and in improving the disease phenotype.

## Results

### Newly designed morpholino oligomers (MOs) against SOD1 effectively reduce protein levels *in vitro*

We designed two 25-nt MO sequences (MO1 and MO2) to target the human SOD1 gene. The MO sequences took into account the best predicted sequence of a bioinformatics tool^27^ and were designed both as Bare MOs (Bare-MOs) and with an octa-guanidine modification (Modified-MOs), which can increase the biodistribution of the MOs *in vivo*. The MOs were synthesized by Gene Tools using a method described elsewhere^20^. A scrambled MO (scr-MO) sequence was designed based on the best control sequence predicted by the bioinformatics tool (Gene Tools, www.genetools.com) and was used in all of the experiments as an internal control.

We performed an *in vitro* analysis of the two lead MO sequences to identify the most efficient one for further *in vivo* evaluation. We tested the Bare-MO sequences (which were suitable for the *in vitro* experiments) in different human cell lines by evaluating the reduction of SOD1 at different time points. We first nucleofected HeLa cells with 20 μg Bare-MOs (MO1 or MO2) or scr-MOs, as suggested by the manufacturer’s protocol. We then used western blot to demonstrate a slight decrease in SOD1 protein levels at all time points with MO1 and a significant reduction (up to 60%) at all time points with MO2 (Supplementary Fig. 1A,B). Following *in vitro* delivery, MO2 sequence was able to silence SOD1 in a human cell type.

### MOs rescue apoptosis-mediated death in fALS induced pluripotent stem cell-derived motor neurons

**Figure 1:**
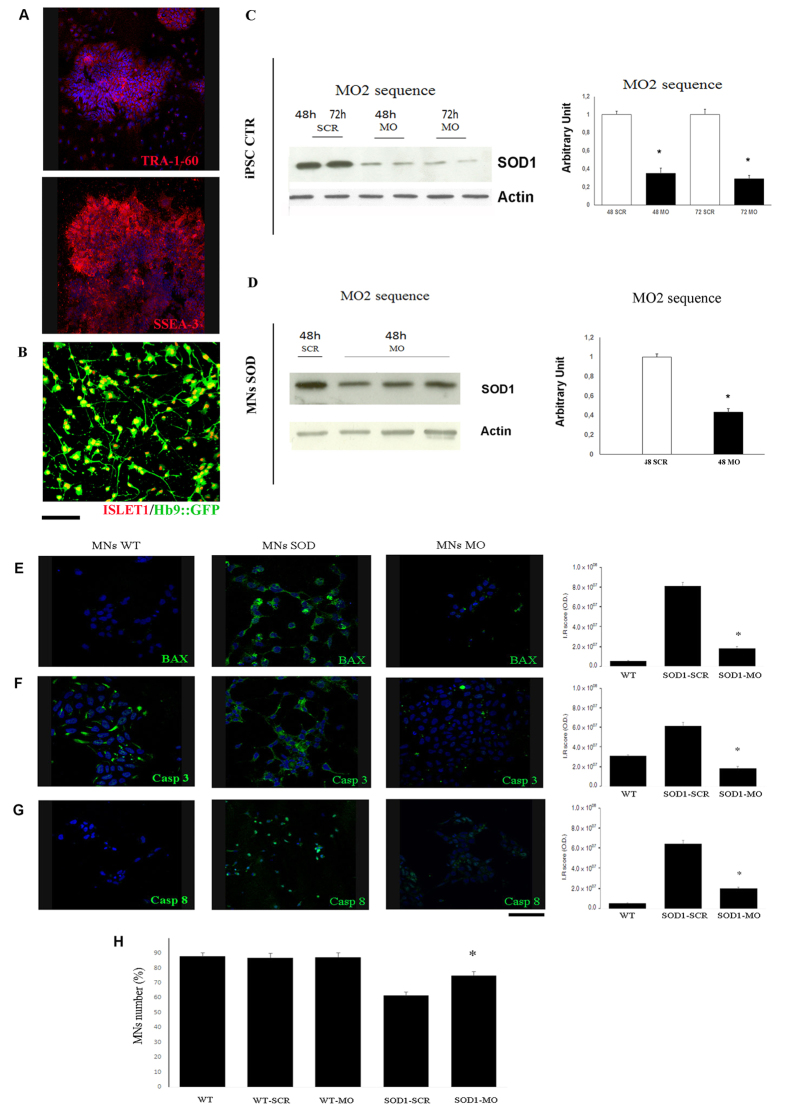
MOs silence of SOD1 protein and rescue apoptosis-mediated death in fALS iPSC-derived MNs. (**A**) Immunocytochemical characterization of iPSC clones derived from human ALS fibroblasts. These iPSCs expressed pluripotency transcription factors including stem cell surface markers, TRA-1–60 (red) and SSEA-3 (red). Blue, 4′,6-diamidino-2-phenylindole (DAPI) nuclear stain. Scale bar: 100 μm. (**B**) After differentiation, iPSCs-derived MNs expressed specific motor neuronal marker, such as Islet-1 (red) and Hb9::GFP (green). Scale bar: 75 μm. (**C**,**D**) Representative western blot (at least three independent experiments for each condition) and quantification analysis of SOD1 protein. Values are means ± SEM of SOD1:β-actin expression levels (*n* = 3), *P < 0.01, ANOVA. SCR: scr-treated cells; MO: MO-treated cells. (**C**) WT iPSCs treated with Bare-MO2 (20 ug) at 48 h and 72 h. (**D**) iPSCs-MNs derived from fALS patient treated with Bare-MO2 (20 ug) at 48 h. (**E**–**G**) Immunocytochemical analysis and immunoreactivity score of iPSC-derived MNs at 30 days in culture demonstrated that the signal of the specific apoptosis markers BAX (**E**), Caspase 3 (**F**) and Caspase 8 (**G**) (all green) is increased in fALS-MNs respect to WT-MNs and is reduced in MO-treated MNs respect to scr-treated samples. Scale bar: 100 μm. Nuclei are stained with DAPI (blue). Quantification of the immunoreactivity score was measured with ImageJ software. *P < 0.01, values are means ± SEM. (**H**) WT-MNs and SOD1-MNs were treated with scr or MO2 at day 0 and counted for the survival analysis at day 1 and after 30 days. The histograms show MNs counted at day 30 and 100% represents the number of MNs counted at day 1 (10.000 cells). MO2 treatment increased survival of the SOD1-MNs compared to SOD1-scr-treated-MNs (*P < 0.01, Values are means ± SEM). No differences between WT-SCR and WT-MO2 treated MNs have been observed, demonstrating the selective protective effect on ALS cells. The histogram shown is representative of three independent trials. WT: healthy MNs; WT-SCR: healthy scr-treated MNs; WT-MO2: healthy MO2-treated MNs; SOD1-SCR: scr-treated MNs; SOD1-MO: MO2-treated MNs.

We previously generated induced pluripotent stem cells (iPSCs) from healthy subject fibroblasts and from fALS-SOD1 patient fibroblasts with a non-viral, non-integrating method^28^,^29^. The iPSCs obtained showed markers of pluripotency and were able to differentiate into MNs (Fig. 1A,B). We tested the most effective MO sequence (MO2) in WT iPSCs (Fig. 1C), and we confirmed a strong decrease in SOD1 protein expression in all conditions (up to 70%). The same result was obtained for iPSC-derived MNs from a fALS patient with a L114P SOD1 mutation^30^ (60% reduction, Fig. 1D). To evaluate the effect of MO treatment on the pathological features of ALS and in particular on the phenotype of ALS-MNs in long-term culture, we examined MN survival in culture at 30 days. We observed a modest, but significant, increase in survival of the fALS MNs treated with MO2 compared to the fALS scr-treated MNs (Fig. 1H; *P* < 0.01). No differences between wild-type (WT)-SCR and WT-MO2 MNs were observed, demonstrating the selective protective effect of MO on ALS cells (Fig. 1H).

Since mut-SOD1 proteins are known to be pro-apoptotic both *in vitro* and *in vivo* and that programmed cell death may be involved in the ALS neurodegenerative process^31^,^32^, we investigated whether the protective effect of MOs on MN survival was correlated with variation in the apoptotic pathway. Interestingly, we observed a reduction of the expression of the apoptotic markers Bax, caspase-3, and caspase-8 in MO2-treated fALS iPSC-derived MNs compared to the fALS scr-treated MNs (Fig. 1E–G) at 30 days in culture.

### Bare-MOs effectively silence SOD1 *in vivo* in SOD1G93A mice

To confirm the results obtained *in vitro*, the two MO sequences were further evaluated *in vivo*, in the B6.Cg-Tg(SOD1-G93A)1Gur/J transgenic ALS mouse model, which carries a high copy number of the mutant human SOD1 allele containing the G93A mutation. The protocols used for the *in vivo* experiments were based on the results obtained in our previous experiments using MOs in an SMA mouse model^25^ and on published results obtained with antisense and shRNA against SOD1^33^,^34^.

**Figure 2:**
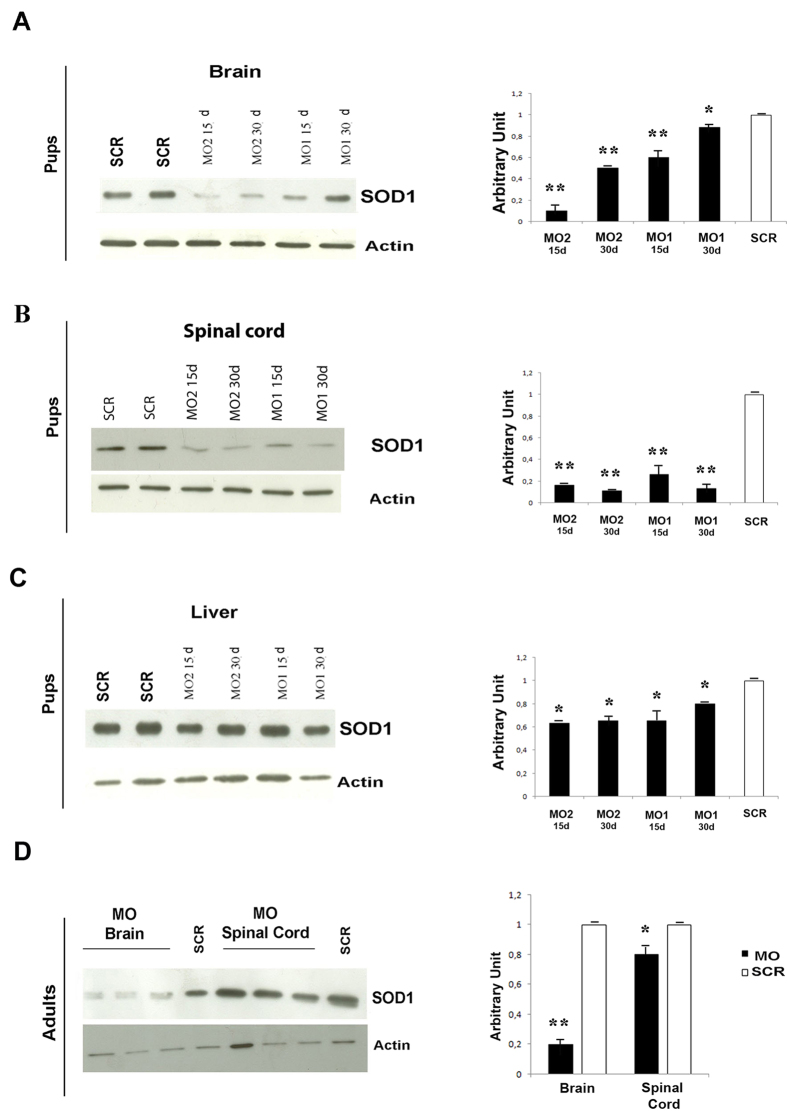
*In vivo* MO treatment silences the SOD1 protein. Western blot and relative quantifications of SOD1 protein in brain (**A**), spinal cord (**B**) liver (**C**) of treated SODG93A pups demonstrated decrease levels respect to scr-treated animals. Mice were treated ICV at P0 (80 μg) and intraperitoneally (IP) at P0, P3 and P6 (80 μg/injections) and tissues were collected 2 or 4 weeks after the treatment. (**D**) Western blot and relative quantifications of SOD1 protein in brain and spinal cord of MO2 treated SODG93A adult mice (ICV at P80, 40 nMoles, tissues harvested 2 weeks after the treatment) demonstrated decrease levels respect to scr-treated animals. The western blots are representative of at least three independent experiments for each condition. Values are means ± SEM of SOD1:β-actin expression levels (*n* = 5), *P < 0.01, **P < 0.001 (ANOVA). SCR: scr-treated mice, MO1: mice treated with Bare-MO sequence 1; MO2: mice treated with Bare-MO sequence 2.

Because the octa-guanidine-modified MOs present a better biodistribution in weaned adult mice, this was the first chemical variant tested. However, the decrease in SOD1 protein in the spinal cords and brains of SOD1 pups treated both locally and systemically was not significant (Supplementary Fig. 2A). This is in accordance with previously described research using octa-guanidine MOs against SMN2^25^. Moreover, systemic injection of Modified-MOs failed to significantly silence SOD1 protein levels in the central nervous system (CNS) of symptomatic SOD1G93A mice (Supplementary Fig. 2B). We observed a toxic effect of intracerebroventricular (ICV) injection in adult mice, both SOD1 mice and WT mice (data not shown), which limits the potential of this route of administration to increase the treatment effects in the CNS. The toxic effects of Modified-MOs were previously described in the literature^23^,^25^. We then tested the efficacy of the two Bare-MO sequences in SOD1 pups, following the injection protocol previously used for MOs in the SMAΔ7 mouse model^25^. At 2 or 4 weeks after the injection, the treatment was completely successful, in both the brain (up to 90% reduction with MO2, Fig. 2A) and spinal cord (between 70% and 90% reduction at all of the time points, Fig. 2B). The SOD1 protein levels were strongly reduced, in peripheral tissues as well (up to 30% reduction, Fig. 2C), confirming the superior efficacy of MO2. Remarkably, Bare-MO2 decreased SOD1 levels by 80% in the brain and by 20% in the spinal cord of adult symptomatic SODG93A mice when injected ICV (Fig. 2D). Overall, we demonstrated the efficacy of the designed Bare-MO sequences, in particular MO2, in decreasing SOD1 protein levels in SOD1 mouse pups and adults.

### MO treatment ameliorates neuromuscular dysfunction and prolongs the survival of SOD1 mice

**Figure 3:**
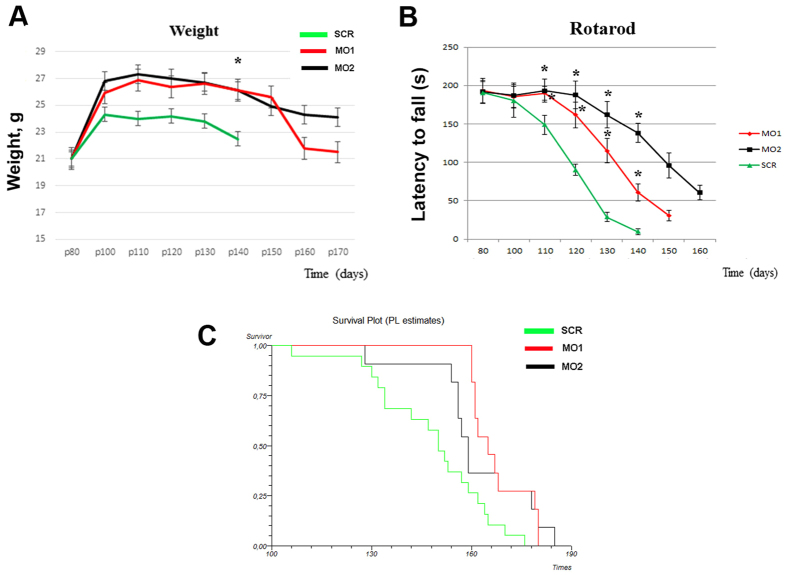
MO treatment improves neuromuscular function and survival in *nmd* mice. SOD1 mice were injected with Bare-MO (40 nMoles) at P85 with ICV injection and intravenously (IV, 12,5 mg/kg) once a week until the end of the disease. (**A**) The mean body weight of MO1 or MO2-treated mice resulted increased relative to scr-treated mice and significantly different at P140 (*P = 0,02, ANOVA, error bars show the SEM). (**B**) Rotarod test data. The performance of MO1 or MO2-treated mice, measured as latency to fall, was significantly increased respect to scr-treated mice (*P < 0.01, one way ANOVA) starting from P110. Error bars show the SEM. (**C**) Kaplan–Meier survival curves of MO1 or MO2-treated mice respect to scr-treated mice (MO1 vs scr *P* = 0,0007, χ^2^ = 11,54; MO2 vs scr *P* < 0,0001, χ^2^ = 16,07). MO1 or MO2-treated mice, n = 11/group; scr-treated mice n = 18.

To assess the effects of MOs on survival and on the neuropathological phenotype of ALS, we treated a cohort of early symptomatic mice with Bare-MOs, combining ICV injection (once, at P80, 40 nMoles) and systemic injection (once per month starting from P80, 12.5 mg/kg) to deliver MO also in peripheral organs. The treatment ameliorated the ALS disease phenotype and increased the survival of SOD1 animals. The growth rates of the mice injected with both MO sequences, as measured by mean body weight, were increased compared to those of the scr-treated mice starting from P100 (Fig. 3A, at P140, *P* = 0.02). To analyze motor function, groups of mice were evaluated with the well-established rotarod test. Starting from P110, the treated animals exhibited significant improvement in rotarod performance relative to the scr-treated mice (Fig. 3B, *P* < 0.01, ANOVA), and some of them were able perform the test after P140, when all of the surviving scr-treated mice could not. Since the rotarod can be affected by the body weight, latency to fall was analyzed also using ANCOVA with body weight as the covariate (data not shown); however the differences were still significant (*P* < 0.05) between the groups. Administration of both MO sequences significantly extended survival, by 19 days or 21 days, compared to the survival of age-matched scr-treated mice (scr-treated mice, 148 ± 17 days; MO1 mice, 167.5 ± 8 days; MO1 vs scr *P* = 0.0007, χ^2^ = 11.54; MO2 mice, 170 ± 11 days; MO2 vs scr *P* < 0.0001, χ^2^ = 16.07, Fig. 3C). Considering both the later weight loss and later decrease in the rotarod performance of the MO-treated mice with respect to the scr-treated mice, the observed increase in survival is likely due to delayed disease onset. With respect to survival and neuropathological phenotype variation, the efficacy of the two MO sequences were comparable, although slightly better results were obtained with MO2, as shown *in vitro*.

### MO-treated mice show MN and axonal protection and a reduction in inflammation

**Figure 4:**
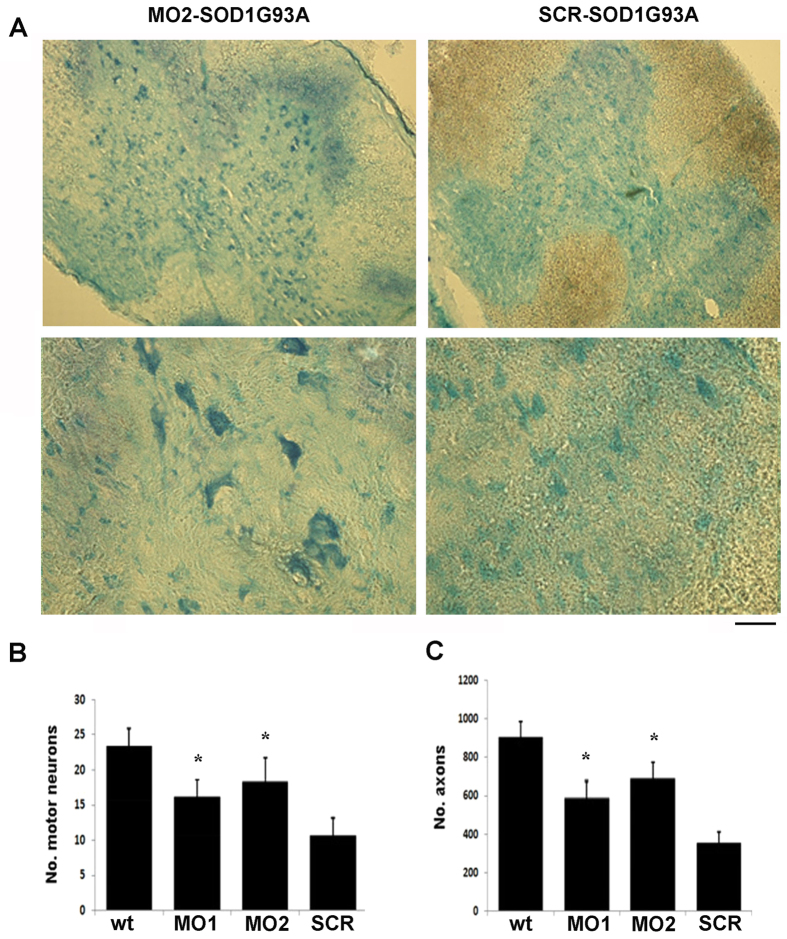
MO treatment increases motor neuron and axon number. Spinal cords were collected at the end stage of the diseases (n = 3/group) (**A**) Representative Nissl stained MN features within the lumbar segment of spinal cord of MO2-treated mice and scr-treated mice. Scale bar: 70 μm (upper panels), 50 μm (bottom panels). (**B**) MN count in the lumbar spinal cord of MOs-treated, scr-treated and WT mice (n = 50 cross sections counted for each mouse, data represent the mean ± SD, *P < 0.01, ANOVA). (**C**) Quantification of myelinated axons in the L4 anterior roots in WT, MOs-treated, and scr-treated mice (the data represent the mean ± SD, *P < 0.01, ANOVA, n = 50 cross sections counted for each mouse). SCR: scr-treated mice; MO1: mice treated with Bare-MO sequence 1; MO2: mice treated with Bare-MO sequence 1, wt: wild-type mice.

To evaluate the impact of MO treatment on the hallmarks of ALS and to understand the mechanisms of the resulting beneficial effects, we analyzed the spinal cords of MO-treated mice and scr-treated mice in terms of neuropathological features. To determine whether SOD1 silencing prevented the loss of MNs, we analyzed the number of MNs in spinal cord sections and ventral spinal nerve roots at the end stage of the disease (Fig. 4A,B, *P* < 0.01). At this stage (P140), we observed a 60% loss of MNs in the scr-treated mice, whereas MN loss was significantly reduced, to 40%, by MO administration.

**Figure 5:**
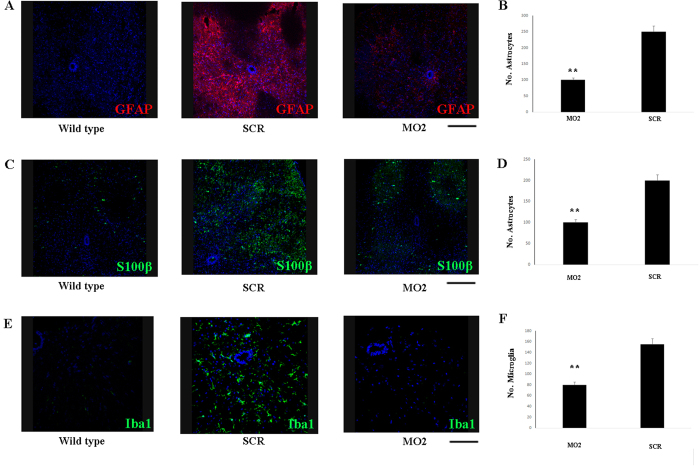
MO treatment reduces micro- and macrogliosis. Expression of GFAP (red) (**A**) and s100β (green) (**C**) in the spinal cord of end stage MO2-treated mice respect to scr-treated mice and WT mice. (**B,D**) Quantification of GFAP and s100β positive cells. MO treatment significantly reduced the presence of astrocytes in the spinal cord of treated mice (**P < 0.001, ANOVA, mean values ± SEM from 5 independent experiments performed in triplicate). (**E**) Expression of Iba1 (green) in the spinal cord of MO2-treated mice respect to scr-treated mice and WT mice. (**F**) Quantification of Iba1 positive cells; MO treatment significantly reduced the presence of microglial cells in the spinal cord of treated mice (**P < 0.001, ANOVA, mean values ± SEM from 5 independent experiments performed in triplicate). Nuclei are stained with DAPI (blue). Scale bar: 70 μm. SCR: scr-treated mice; MO2: mice treated with Bare-MO sequence 2.

We next examined the neuroprotective effects of the treatment by counting the number of axons in the L4 ventral root and found an increase of 35% in the MO2-treated animals with respect to the scr-treated animals (Fig. 4C, *P* < 0.01). Many studies have demonstrated the presence of an inflammatory response in ALS patients and in mutated SOD1 mice, suggesting the involvement of reactive astrogliosis and microglia in the disease pathogenesis (for a review, see^35^). Astrocytes modify their morphology, adopting hypertrophic nuclei and cell bodies with distinct, long, and thick processes with exhibiting increased expression of glial fibrillary acidic protein (GFAP), which is the main constituent of the intermediate filament system of adult astrocytes. The morphological and molecular modifications of astrocytes and microglia are associated with a shift from a neuroprotective to a neurodegenerative role towards MNs. Therefore, we analyzed whether a change in inflammatory reactions and astrogliosis and microglia occurred after MO treatment in lumbar spinal cords. Immunohistochemistry using the astrocyte markers GFAP and s100β demonstrated a sharp reduction in astrogliosis and activated microglia in the MO-injected animals compared to the scr-treated mice (Fig. 5A–D, *P* < 0.001). Moreover, we detected a remarkable reduction in the expression of Iba-1, a marker of activated microglia, in the MO-injected animals (Fig. 5E,F; *P* < 0.001). Overall, the sharp decrease of astrogliosis and microglial activation likely accounts for the protection of MNs and axons and, consequently, for the observed functional improvement.

### MO treatment improves the size of myofibers and ameliorates defects in skeletal muscle

SOD1G93A mice develop muscle atrophy, severe myopathy, and myofiber cell death. H&E-stained sections of quadriceps muscle demonstrated that the treatment positively affected degenerating skeletal muscle by improving myofiber morphology and organization and reducing the irregular connective tissue infiltrates detected in the quadriceps harvested from scr-treated mice (Supplementary Fig. 3).

### MO reduces misfolded SOD1 protein

**Figure 6:**
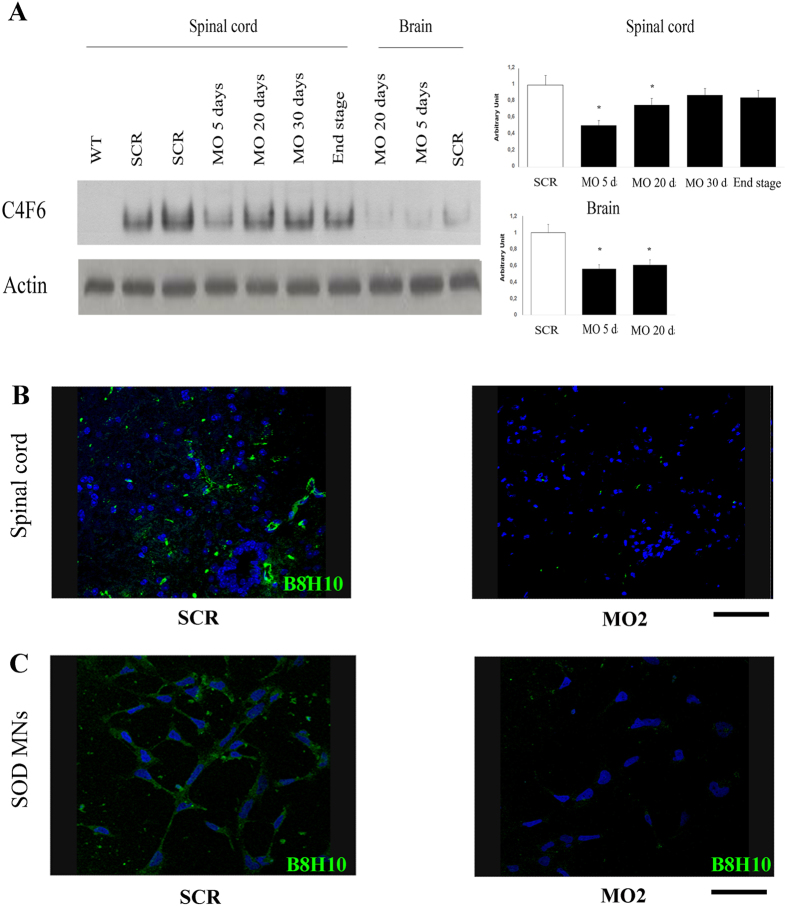
MO reduces the misfolded SOD1 protein. (**A**) Non-denaturing gel with misfolded specific antibody C4F6 demonstrates decrease levels of misfolded SOD1 protein in brain and in spinal cord of MO2 treated SODG93A adult mice (ICV, 40 nmoles, at P80) respect to scr-treated animals at different time-points. Denaturing gel for β-actin served as loading controls. WT: wild-type mice; SCR: scr-treated SODG93A mice; MO: MO-treated SODG93A mice. Experiment shown is representative of three independent trials. (**B**) Expression of misfolded specific antibody B8H10 (green) is reduced in the spinal cord of MO2-treated mice respect to scr-treated mice. Scale bar: 70 μm. Nuclei are stained with DAPI (blue). SCR: scr-treated mice; MO2: mice treated with Bare-MO sequence 2, WT: wild-type mice. (**C**) The reduction is confirmed also *in vitro* in MO-treated fALS iPSC-derived MNs. Scale bar: 100 μm. Nuclei are stained with DAPI (blue). SCR: scr-treated mice; MO2: mice treated with Bare-MO sequence 2, WT: wild-type mice.

SOD1 can exist in an altered/abnormal conformation, referred to as misfolded SOD1, which is associated with the inherent toxic nature of SOD1 mutations^10^,^36^,^37^,^38^. A number of antibodies targeting misfolded SOD1 have recently been developed^13^,^14^,^39^. We tested two antibodies specific for the misfolded form of SOD1, the clone B8H10 and the clone C4F6, to investigate whether MOs act by reducing the expression of this non-native conformation of SOD1. We analyzed misfolded SOD1 levels using a non-denaturating gel in MO2-treated brains and spinal cords that were collected at different time points and compared them to the levels in scr-treated mice. We observed strong expression of the altered SOD1 protein conformation in all of the SODG93A samples and a reduction in its expression at all times point after MO treatment (Fig. 6A). The decrease was confirmed by immunohistochemistry of the lumbar spinal cords of SOD1G93A scr-treated mice and SOD1G93A MO-treated mice (Fig. 6B). Misfolded SOD1 protein accumulated in fALS iPSC-derived MNs as well, and this accumulation was reduced after treatment (Fig. 6C). These preliminary data suggest that misfolded SOD1protein may be a therapeutic target and that MOs can reduce levels of misfolded SOD1 both *in vitro* and *in vivo*.

## Discussion

In the current study, we have shown that MO-mediated silencing of mutant SOD1 expression in the CNS reverses an ALS disease phenotype in a transgenic mouse model, providing evidence of the remarkable therapeutic potential of this approach. Currently, there is a critical need for new and effective treatments for ALS. In fact, only one drug, Riluzole, is approved by the FDA as an ALS treatment, and Riluzole exhibits only a modest effect on survival (3-month increase in median survival)^40^. Given the accumulating data on the pathogenetic role of SOD1 in ALS, the development of therapeutic strategies has been focused on silencing mutant SOD1 protein to reduce its toxicity. Unfortunately, these strategies remain in the early stages. Antisense oligonucleotides and virus-delivered RNA interference have been investigated in rat and mouse ALS models^33^,^34^,^41^,^42^,^43^,^44^ with promising outcomes. However, many of these approaches were used to achieve SOD1 knockdown before disease onset. Furthermore, they present technical difficulties or safety concerns that hamper their further development towards application in patients. Nevertheless, direct cerebrospinal fluid infusion of antisense oligonucleotides against SOD1 has been recently explored in a clinical trial^21^, demonstrating the tolerability and safety of the approach. Unfortunately, a limited reduction of SOD1 levels was obtained due to the low dosages used for safety reasons. Here, we exploited the peculiar chemistry, known stability, effectiveness, safety, and wide tissue distribution of MOs^19^,^25^,^26^ in the context of ALS. Due to these features, the MO approach promises to be superior to conventional phosphorothioate oligonucleotides. Compared to a viral approach, the use of MOs has a more attractive safety profile and the distinct advantage that the effects can be reversed by stopping the administration.

We demonstrated that local and systemic delivery of MOs effectively reduced SOD1, ameliorated pathological hallmarks, reduced disease duration prolonging survival in ALS rodents. We hypothesize that the decreasing of SOD1 levels peripherally can contribute to the observed phenotype amelioration. We further demonstrated the efficacy of the MO therapy in a human disease model, using human fALS iPSC-derived MNs. Interestingly, the MNs derived from our fALS iPSC line exhibited decreased numbers in culture compared to WT MNs; this reduction was associated with an increase in apoptotic marker expression, in agreement with Kiskinis *et al.*^31^. MO treatment was able to reverse these two pathological characteristics. Several studies support the view that apoptosis plays a key role in the neurodegenerative processes of both fALS and sALS. Indeed, increased expression of pro-apoptotic proteins, such as Bax, Bad, and activated caspases, has been detected in the spinal cord in transgenic models of fALS as well as in human ALS patients, with or without SOD1 mutations (for a review, see^32^). One of the beneficial effects of MO may be due to the inhibition of apoptosis, although further studies are needed to investigate the exact mechanism of action.

We have achieved one of the most impressive reductions of SOD1 (up to 80% reduction) ever reported using a treatment in the ALS SOD1G93A mouse model after disease onset, which corresponds to the period when ALS is usually diagnosed in patients. These data represent a proof of concept that SOD1 silencing after disease onset, during clinically relevant points in the disease process, can be beneficial in rapid, progressive models of ALS, and demonstrate the promise of MO delivery during a symptomatic phase in terms of efficacy.

We delivered a single local injection of MOs in CNS, the effects of which usually lasted for a month. Thus, even more pronounced positive effects on survival and SOD1 reduction can be expected using continuous delivery with a mini-pump (which are clinically available) or with repeated administration.

The MO treatment resulted insubstantial protection of MNs and axons in the SOD1 mice. However, it is becoming increasingly clear that non-neuronal cells in the spinal cord also contribute to initial toxicity and disease progression in ALS^35^. In particular, the neuroinflammatory processes produced by microglia and astrocytes appear to be an important pathological hallmark of ALS pathology, as demonstrated in ALS patients and mutated SOD1 mice^35^. One of the advantages of the MO approach, with respect to other therapeutic strategies, is that it allows SOD1 targeting in both neuronal and non-neuronal cells in the CNS and can thus act on both the cell-autonomous and non-cell autonomous pathogenic mechanisms of ALS. One of the most striking effects of MO injection in the ALS mice was the significant reduction in astrogliosis and microgliosis, which is likely responsible for the treatment effectiveness after disease onset.

Glial cells are activated early in ALS, in both the human and rodent CNS, suggesting that neuroinflammation plays an important role in the cascade of events that trigger the death of MNs. Because these processes also occur in sALS, MO-mediated reduction of reactive astrocytosis and microgliosis is promising from a therapeutic perspective, especially when the ability of MOs to ameliorate MN survival by reducing apoptotic events is also considered. Moreover, broadly targeting regions of the brain and spinal cord enabled significant improvement of neuromuscular function, as demonstrated by improved muscle trophism and an increase in lifespan.

Translating these positive results to human cases of ALS, primarily SOD1fALS, is relatively feasible. Indeed, in our study, we demonstrated that MO-mediated reduction of SOD1 is safe and well tolerated in ALS rodent models, with an absence of adverse effects. Previous data involving transgenic mice have suggested that lowering endogenous SOD1 activity is safe^33^, and the recent clinical trial with the ASO ISIS 333611 confirms this notion in humans as well^21^. Moreover, the local delivery method used in our study can be performed in humans via local intrathecal injection, a clinically common procedure that was recently shown to be safe in the Phase 1 ISIS 333611 clinical trial^21^. The non-invasive mode of vector delivery used in this study (local and intravenous) and the substantial efficacy achieved demonstrate the enormous and realistic potential to advance MO approaches to the clinic.

SOD1 can exist in an altered/abnormal conformation, referred to as misfolded SOD1, that is associated with the inherent toxic nature of SOD1 mutations^10^,^36^,^37^,^38^. In our study, we demonstrated that MO is able to decrease the misfolded form of SOD1 in the CNS of treated mice and in fALS iPSC-derived MNs *in vitro*. This result is extremely important because several reports have suggested that SOD1 misfolding is involved in sALS^9^. However, whether SOD1 is involved in sporadic disease remains controversial. In sALS, WT SOD1 can assume aberrant conformations that resemble those of mutant SOD1 in ALS; misfolded WT SOD1 can reproduce some of the cytotoxicity triggered by mutant SOD1^10^,^11^,^12^. Importantly, these findings indicate that in a proportion of sALS patients, therapies aimed at reducing SOD1, including an MO-based approach, may be effective.

MO-based strategy can be applied to target other pathological molecular pathways in the symptomatic phase of ALS to achieve a therapeutic effect. Furthermore, MO silencing of a mutated gene may be equally applicable to other dominantly inherited neurodegenerative disorders related to a toxic gain-of-function, such as α-synuclein-associated inherited forms of Parkinson’s disease or Huntington’s disease, in which depletion of the endogenous pathological protein could be curative.

Our data represent the first demonstration of the therapeutic efficacy and feasibility of MO-mediated gene silencing for ALS, both fALS and sALS, and for other neurodegenerative diseases. Moreover, our *in vitro* and *in vivo* experiments in murine and human models provide an excellent rationale for clinical translation to patients.

## Materials and Methods

### Morpholino oligomers

The MO sequence number 1 was GCACGCACACGGCCTTCGTCGCCAT and the number 2 was CACAGGCCTTCGTCGTCGCCATAACTC. These two MO sequences were synthesized as Bare-MO without any modifications or with an octa-guanidine modification (Gene Tools). The scr-MO sequences were designed based on the best control sequence predicted by the bioinformatic tool (Gene tools, www.gene-tools.com). MOs were dissolved in sterile saline solution at the appropriate concentration for injection.

### Cell culture

iPSC lines were reprogrammed from fALS fibroblasts (L114P SOD1 mutation^30^) and healthy subjects (from Eurobiobank with appropriate ethical approval), using a non-viral protocol, as previously described^29^. To differentiate iPSCs into spinal MNs, we followed a multistep protocol developed for human embryonic stem cells (ESCs) and iPSCs^28^. Cells were plated with neuronal medium (Dulbecco’s modified Eagle’s medium (MEM)/F12 (Life Technologies), supplemented with MEM nonessential amino acids solution (Life Technologies), N2 (Life Technologies), and heparin (2 mg/ml; Sigma- Aldrich), to which retinoic acid (0.1 μM; Sigma-Aldrich) was added after 10 days for neural caudalization. Posteriorized neuroectodermal cells were collected at day 17 and suspended for a week in the same medium with retinoic acid (0.1 μM) and Sonic hedgehog (100–200 ng/ml; R&D Systems), followed by the addition of BDNF and GDNF on day 24 (10 ng/ml; PeproTech). A centrifugation gradient was used to maximize motor neuron acquisition^28^. Cells were fixed and immunostained for quantification following seeding in culture for at least 24 h.

### Immunocytochemistry of iPSCs and their derivatives

Cells were fixed in 4% paraformaldehyde for 10 min, blocked with 10% BSA and permeabilized with 0.3% Triton X-100 in phosphate-buffered saline (PBS). We incubated the cells with primary antibodies for the pluripotency marker TRA1-60, SSEA-3 (1:100, BD, Cy3 conjugated), for the motor neuronal marker SMI32 (1:200; mouse, Millipore) or for apoptotic markers Bax (1:100, rabbit, Cell Signaling) Caspase-3 (1:100, rabbit, Cell Signaling) Caspase-8 (1:100, rabbit, Santa Cruz Biotechnology) overnight and with secondary antibodies anti-rabbit, anti-mouse FITC-conjugated (Dako, 1:100) for 1 h. For all imaging, we used a confocal LEICA LCS2 microscope. We quantified immunoreactivity score for apoptotic markers measuring the optical densities of each staining with ImageJ software at 30 days after the treatment.

### MO transfection and MNs count *in vitro*

Hela and iPSCs were electroporated with the Neon Nucleofection System (Life Technologies) following the MO manufacturer instructions. Briefly, cells were trypsinized, counted and washed with PBS by centrifugation. The pellet was resuspended in 100 ul Resuspension Buffer R and 20 ug MO were added. The cell-MO mixture was aspirate with the Neon™ Pipette and electroporated with the set pulse conditions on the device. Transfected cells were transfer on the prepared culture plate containing pre-warmed medium. Cells were harvested 48 h and 72 h after nucleofection. For MNs count: cells were treated with MO2 at day 0 and counted for survival analysis at day 1 and after 30 days. We quantified MNs using identification of Hb9::GFP^29^, counting 10 randomly selected fields/well (3 wells per condition per experiment in five experiments).

### Animal procedures

All transgenic animals were purchased from The Jackson Laboratory. All animal experiments were approved by the University of Milan and Italian Ministry of Health review boards, in compliance with US National Institutes of Health Guidelines^45^. We used transgenic mice of the strain B6.Cg-Tg(SOD1-G93A)1Gur/J, which carries a high copy number of the mutant human SOD1 allele containing a Gly93Ala (G93A) substitution. Progeny for experimental analyses was obtained by breeding SOD1G93A transgenics with C57BL/6 wild-type mice. Transgenic mice were identified by polymerase chain reaction, as previously described^45^.

The Modified-MO was tested in adult mice with a systemic injection as recommended by the manufacturer. We treated symptomatic SOD1G93A transgenic mice (P120) with 12.5 mg/kg of Modified-MO administered IV twice in a week. Tissues were harvested the day after the second treatment. The Bare-MO and the Modified-MO were tested in pups, by injecting ICV at P0 (80 μg) and intraperitoneally (IP) at P0, P3 and P6 (80 μg/injections) in SOD1 mice and we collected tissues 2 or 4 weeks after the treatment for western blot analysis^25^. For western blot analysis Bare-MO2 was injected ICV in adult SOD1 mice at P80, 40 nMoles and the tissues harvested 2 weeks after the treatment.

For the survival analysis adult SOD1 mice were injected with Bare-MO (40 nMoles) at early symptomatic phase (at P85) with ICV injection follow the standard stereotactic coordinate and intravenously (IV, 12,5 mg/kg) according to Gene Tools instructions, once a week until the end of the disease.

### Western blots

Western blot analysis was performed as previously described^25^. Briefly, frozen spinal cord specimen were homogenized in RIPA-buffer (50 mM Tris pH 7.5, 150 mM NaCl, 1% NP-40, 0.5% sodium deoxycholate and 0.1% SDS) supplemented with protease inhibitor cocktail tablets (Roche) and the protein content was measured using Lowry assay. An amount of 40 μg of total protein was boiled in Laemmli buffer for 5 min and separated on a 8% sodium dodecyl sulfate–polyacrylamide gel electrophoresis SDS–PAGE. After electrophoresis, proteins were transferred on a nitrocellulose membrane, blocked in 5% BSA (for 1 hour at room temperature) and incubated with a primary anti-SOD-1 antibody (1:1200, R&D) overnight at 4 °C. The day after the nitrocellulose membrane was incubated with anti-rabbit IgG conjugated to horseradish peroxidase (1:2700). Finally, the immune complexes were detected using chemiluminescent detection reagents. The nitrocellulose membrane was stripped and reprobed with anti-actin monoclonal antibody for equal loading control. For the quantification analysis of all western blot, the values showed were means ± SEM of SOD1:β-actin expression levels (*n* = 3).

### Western blots for SOD misfolded protein

Samples for native western blot analysis were prepared in 1X loading buffer ph 8.6 by sonication, 30 ug total protein were separated by PAGE with 12% Tris-Glycine gels in Tris-Glycine buffer at 4 °C. After 15 min of incubation in 0,1% SDS the proteins were transferred to Nitrocellulose in Transfer buffer pH 9.2 for 90 min at 4 °C. Membrane were air dry fixed and blocked for 1 hour at 25 °C in 5% non fat dry-milk , probed with C4F6 overnight at 4 °C followed by HRP-coniugated rabbit anti-Mouse IgG and visualized by chemiluminescence.

### Neuromuscular evaluation and survival

All groups of SOD mice were monitored daily after treatment with MOs sequence or scramble for phenotypic hallmarks of disease. The investigators that executed the functional assessment were blind to the treatment. Body weight was recorded weekly, and motor function was tested weekly with an accelerating rotarod device (4–40 rpm; Rota-Rod 7650; Ugo Basile). We recorded the length of time that each mouse remained on the rotarod. Mortality was scored as the age at death. The animals were sacrificed when they were unable to right themself within 30 s when positioned in a supine position^29^. The examinators were blinded to the treatments.

### Spinal cord analysis

Animals were euthanized at the end stage, perfused and fixed with 4%PFA in PBS (pH 7.4). The spinal cord was isolated, immersed in PFA for 1 hand then soaked overnight in 20% sucrose in PBS (pH 7.4). The fixed tissue was frozen in Tissue Tek OCT compound (Sakura) with liquid nitrogen. The tissues were cryosectioned and mounted on gelatinized glass slides. Every tenth 20 mm section was collected. All sections were blocked with 10% BSA and permeabilized with 0.3%Triton X-100in PBS. Sections were processed for multiple markers to determine inflammatory reaction. Primary antibodies were added overnight at 4 °C GFAP (1:200, mouse monoclonal Cy3 conjugate, Sigma), S100β (rabbit, 1:3000, Abcam), Iba-1 (rabbit, 1: 1500, Wako) and B8H10 (1:100, mouse, Medimabs). As secondary antibody anti-rabbit, anti-mouse FITC-conjugated (Dako, 1:100) for 1.5 h at RT was used. Histological analysis for quantification of microglia and astrocytes was performed as described^46^, on an average of six sections of the lumbar spinal cord for each animal in each group were counted.

### *In vivo* motor neuron and axon count

A group of mice was used for the histopathological analysis (n = 3). The lumbar spinal cord region was processed for paraffin embedding. Serial cross sections (12 μm thick) of the lumbar spinal cords were made, and every fifth section was processed and Nissl stained, as reported previously^47^. The number of all MNs counted in these cross sections (n = 50 for each mouse) were analyzed. The sections were analyzed at 20× magnification in the anterior horn (either left or right) for the presence of all neurons in that region. All cells were counted within the ventral horn below an arbitrary horizontal line drawn from the central canal. Only neuronal cells with at least 1 nucleolus located within the nucleus were counted, as previously described^47^.

The axonal count was performed as previously described on semi-thin transverse sections stained with toluidine blue^48^. L4 lumbar anterior roots were examined for axon counting on the optic microscope at 60× magnification.

### Morphological analysis of the muscles

A group of mice was used for histological analysis of the periphery (n = 3). The nitrogen frozen quadriceps of the end-stage MO-treated, scr-treated or WT mice were cryosectioned and stained for H&E to determine myofiber dimension as reported^25^. Approximately 500 non-overlapping myofibers from each muscle were randomly selected and photographed at 40× magnification.

### Statistical analysis

We used StatsDirect for Windows (version 2.6.4) for all analyses, and the null hypothesis was rejected at the 0.05 level. All counting data from immunocyto/histochemical analyses and cell survival were expressed as mean values ± SEM and statistically evaluated by one-way ANOVA followed by Tukey’s post-hoc analysis. We analyzed differences in the results of morphometric axonal length measurement (mean ± SEM for five independent experiments) with the Kolmogorov–Smirnov test (http://www.physics.csbsju.edu/stats/KS-test.n.plot_form.html). Kaplan–Meier survival analysis and the log-rank test were used for survival comparisons. The growth curve and rotarod results were analyzed by ANOVA followed by a Tukey’s post-hoc analysis for multiple comparisons. Latency to fall was analyzed also using ANCOVA with body weight as the covariate.

## Additional Information

**How to cite this article**: Nizzardo, M. *et al.* Morpholino-mediated SOD1 reduction ameliorates an amyotrophic lateral sclerosis disease phenotype. *Sci. Rep.* **6**, 21301; doi: 10.1038/srep21301 (2016).

## Supplementary Material

Supplementary Information
