# Supplementary material for: Morpholino-mediated SOD1 reduction ameliorates an amyotrophic lateral sclerosis disease phenotype
Source: Sci Rep. 2016 Feb 16;6:21301. doi: 10.1038/srep21301 (PMC4754711; doi:10.1038/srep21301)
Supplement: Supplementary Information [file srep21301-s1.pdf]

Morpholino-mediated SOD1 reduction ameliorates an amyotrophic lateral sclerosis disease phenotype

M. Nizzardo[1*], C. Simone[1], F. Rizzo[1], G. Ulzi [1], A. Ramirez[1], M. Rizzuti[1], A. Bordoni[1], M. Bucchia[1], S. Gatti[2], N. Bresolin[1], G. P. Comi[1], and S. Corti[1]

Supplementary Information

Supplementary Figures

Fig. S1. Validation of MO sequences in vitro

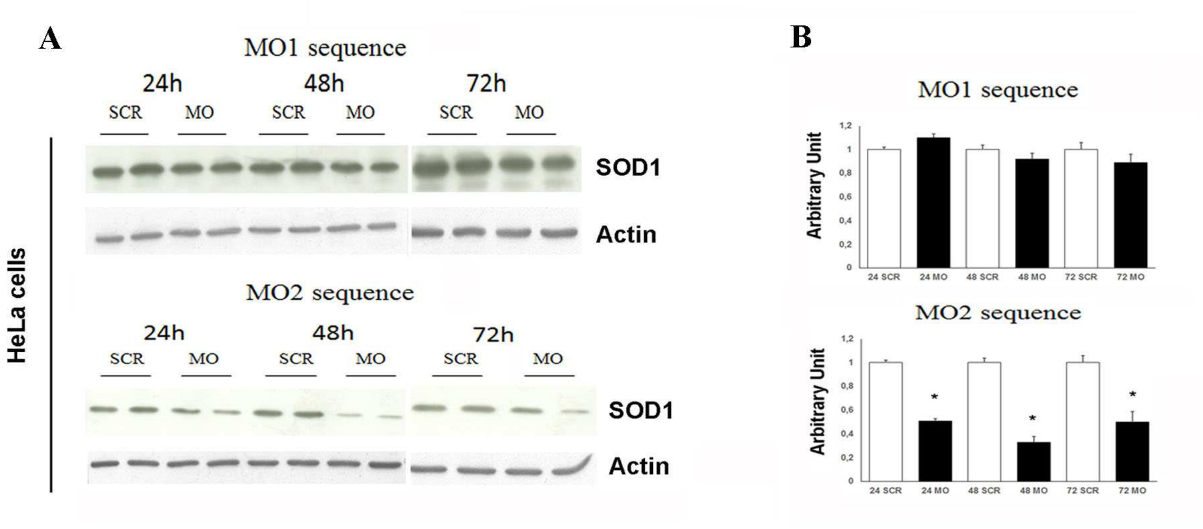

(A) Western blot analysis of SOD1 level at 24, 48 and 72 h after transfection of HeLa cells with MO1, MO2 or scr-MO (20ug/each). **(B)** Quantification analysis of SOD1 levels in MO1 or MO2 transfected HeLa cells respect to scramble transfected. The western blots are representative of at least three independent experiments for each condition.

SCR: scr-treated cells, MO: cells treated with Bare-MO sequences. Values are means ± SEM of SOD1:β-actin expression levels (*n* = 3), *$P < 0.01$.

Fig. S2. Systemic injection of Modified-MO fails silencing of SOD1 protein

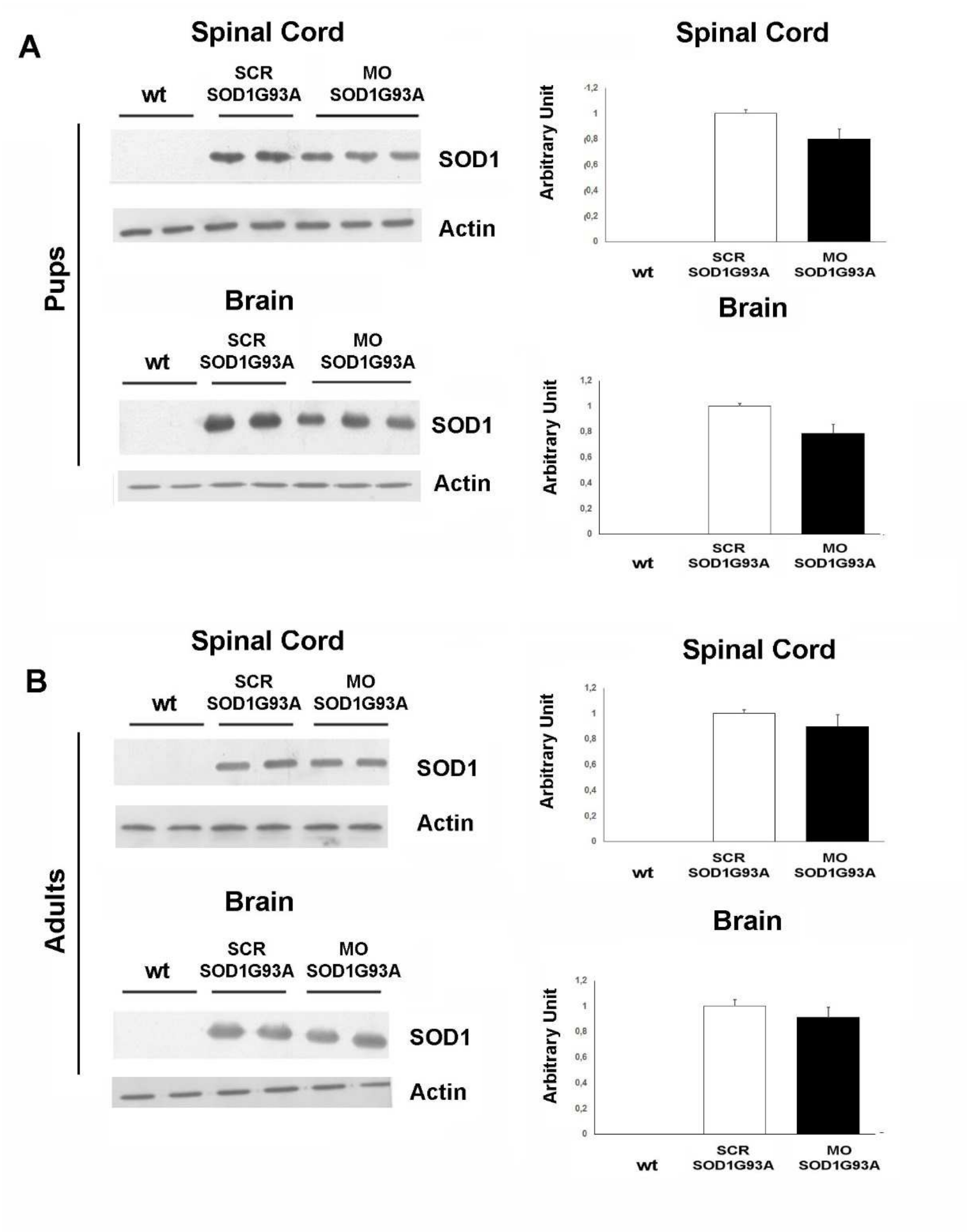

(A) Western blot and quantification analysis of SOD1 level in spinal cord and brain of pups treated both ICV (80ug, P0) and IP (at P0, P3 and P6, 80ug/injections) with Modified-MO. Tissues collected 2 or 4 weeks after the treatment. **(B)** Western blot and quantification analysis of SOD1 level in spinal cord and brain of adult mice treated systemically (at P120, IV, 12.5 mg/kg twice in a week) with Modified-MO. Tissues harvested the day after the second treatment.

Values are means ± SEM of SOD1:β-actin expression levels (*n* = 3). The western blots are representative of at least three independent experiments for each condition. SCR: scr-treated mice; MO: mice treated with Modified-MO, wt: wild-type mice.

Fig. S3. MO treatment improves the size of myofibers in skeletal muscle of treated mice

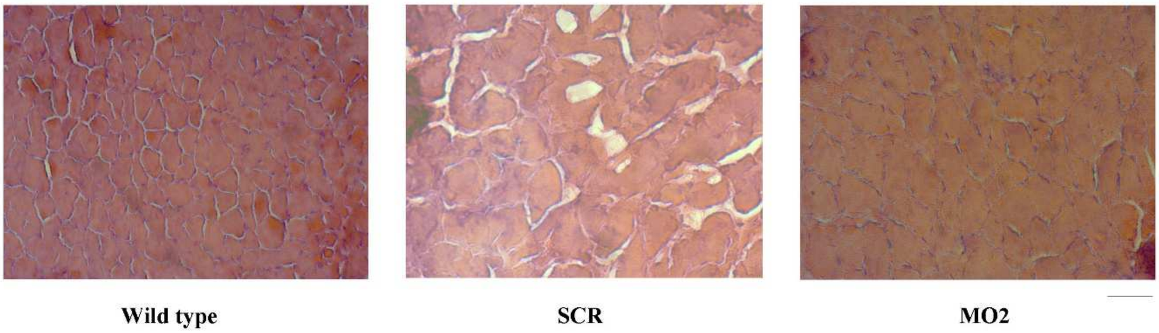

Representative images of quadriceps at the end stage of disease (n=3/each group) stained with haematoxylin and eosin of WT, scr-treated SOD1 mouse and MO2-treated SOD1 mouse. Approximately 500 non-overlapping myofibers from each muscle were randomly selected and photographed at 40× magnification. The images shown are representative of three independent analysis.

Scale bar: 100 μm. SCR: scr-treated mouse, MO2: MO2-treated mouse.
